# TrkB promotes laryngeal cancer metastasis via activation PI3K/AKT pathway

**DOI:** 10.18632/oncotarget.21711

**Published:** 2017-10-09

**Authors:** Liang Jiang, Zhihai Wang, Chuan Liu, Zhitao Gong, Yucheng Yang, Houyong Kang, Yanshi Li, Guohua Hu

**Affiliations:** ^1^ Department of Otolaryngology, The First Affiliated Hospital of Chongqing Medical University, Chongqing, 400016 China; ^2^ Department of Otolaryngology Head and Neck Surgery, Affiliated Hospital of Southwest Medical University, Luzhou, 646000 China

**Keywords:** TrkB, laryngeal cancer, PI3K/AKT pathway, tumorigenicity

## Abstract

**Objectives:**

The aim of our study was to investigate the role of TrkB pathway in tumor occurrence and development for in order to provide theoretical basis to laryngeal cancer therapy.

**Materials and methods:**

Biological characteristics of the cells were studied by migration tests and colony forming assay. Gene and protein expression analysis was performed by RT-PCR or western blot. *in vivo* experiments were conducted in syngeneic BALB/c mice.

**Results:**

Significant changes in protein and gene expression, including higher expression level of TrkB, were found in cells and laryngeal cancer specimens. we demonstrated that TrkB activates AKT via c-Src, leading to increased proliferation. Also, TrkB induced EMT via increased expression of EMT related transcription factors such as Twist-1 and Twist-2.

**Conclusion:**

Our data indicate TrkB are overexpressed in laryngeal cancer, and TrkB signaling is involved in tumorigenicity of laryngeal cancer. These observations suggest that TrkB is a promising target for future intervention strategies to prevent tumor metastasis, EMT program in laryngeal cancer.

What is already known about this subject?

• Cancer of the larynx is one of the most common types of head and neck cancer.

• The survival rate of advanced laryngeal cancer is only 30 to 40%.

• The tropomyosin-related kinase B receptor (TrkB), together with TrkA and TrkC, are neurotrophin receptors regulating the proliferation and differentiation of neuronal cells.

What are the new findings?

• TrkB are overexpressed in laryngeal cancer.

• TrkB signaling is involved in tumorigenicity of laryngeal cancer.

• TrkB acts as a key regulator of the PI3K/AKT signal pathway-mediated tumor metastasis.

How might these results change the focus of research or clinical practice?

• These observations suggest that TrkB is a promising target for future intervention strategies to prevent tumor metastasis, EMT program in laryngeal cancer. Our study provides molecular insight into the tumor metastasis and has important implications in elucidating oncogenic processes.

## INTRODUCTION

Cancer of the larynx is one of the most common types of head and neck cancer. Laryngeal cancer is also the second most common tumor of the respiratory tract and is the eleventh most common cancer worldwide with high mortality rate. Among them, the laryngeal squamous cell carcinoma accounts for approximately 95% of all malignant tumors of the larynx [1]. Laryngeal cancer often occurs in middle-aged and elderly men with an estimated incidence rate of 5.8 cases per 100,000 cases and the mortality in laryngeal cancer for males is 2.2/100,000. According to the data from National Cancer Center of China, it was estimated that there were 26,400 new cases diagnosed as laryngeal cancer in 2015, including 23,700 males and 2,600 females. Moreover, the numbers of mortality were 14,500 cases with 12,600 males and 1,900 females [2]. Although new surgical approaches, more chemotherapy drugs and advanced radiotherapy have been used in the treatment of laryngeal cancer, unfortunately, the 5-year survival rate of laryngeal cancer has decreased from 66% to 63% over past 40 years [3]. The survival rate of advanced laryngeal cancer is only 30 to 40%. The main reason is that the lack of definite diagnosis and the resistance to chemo-/radiotherapy will cause the high recurrent rate, reduce the efficacy of treatment and increase the mortality rate. Therefore, it is very necessary to fully understand the underlying molecular mechanisms and to investigate more effective diagnostic and therapeutic targets [4].

Several risk factors have been implicated in the pathogenesis of laryngeal cancer, including tobacco use, alcohol consumption, air pollution, genetics, nutrition, bacterial infection, life-style and occupational factors [5–9]. Moreover, more and more host genes, non-coding RNA targeting genes, transcription factors and enzymes have been involve in the whole complex processes of tumorigenesis and development of laryngeal cancer [10]. Among them, tropomyosin-related kinase B receptor (TrkB), together with TrkA and TrkC, are neurotrophin receptors regulating the proliferation and differentiation of neuronal cells [11]. TrkB is a 145-kDa receptor tyrosine kinase which can be activated by brain-derived neurotrophic factor (BDNF) and neurotrophin 4 (NT4). The altered TrkB expression, signaling and mutations have been proved to play an essential role in regulating oncogenesis and tumor progression in various cancers, including carcinomas of the pancreas, lung, colon and prostate, neuroblastoma and multiple myeloma [12]. Increasing evidences have suggested that TrkB is essential for tumor progression such as invasion, metastasis, angiogenesis, and resistance against therapeutic agents, however, the underlying mechanism remains unclear [13].

The phosphoinositide 3-kinase (PI3K) signaling pathway is the most commonly mutated pathway in head and neck squamous cell carcinoma [14]. Activated AKT mediated anti-anoikis and apoptosis *via* mitochondrion-driven apoptotic pathway, rendering cancer cells survival, chemoresistance and invasiveness [15]. Previous studies have shown that the activation of TrkB by BDNF could mediate the anoikis suppression and metastasis by activating PI3K/AKT pathway [16]. The overexpression of TrkB has been reported in different malignant tumors, such as neuroblastoma, pancreatic cancer, breast cancer, lung cancer, prostate cancer and myeloma [12]. However, the relationship between the expression of TrkB and prognosis in laryngeal cancer has not been fully understood. Moreover, the signaling mechanisms of TrkB-mediated tumorigenesis and epithelial-to-mesenchymal transition (EMT) of laryngeal cancer are still unclear. Therefore, in this report, we identify a signaling network present in laryngeal cancer cells that is regulated and coordinated by TrkB. We found that TrkB was overexpressed in human laryngeal cancer and acted as a key regulator of the PI3K/AKT signal pathway-mediated tumor metastasis. Our study provides molecular insight into the tumor metastasis and has important implications in elucidating oncogenic processes of laryngeal cancer.

## RESULTS

### The expression of TrkB significantly increases in laryngeal cancer

Immunohistochemical assay was performed to investigate the protein expression of TrkB in 69 carcinomas and 32 tumor-adjacent normal laryngeal tissues.

**Figure 1:**
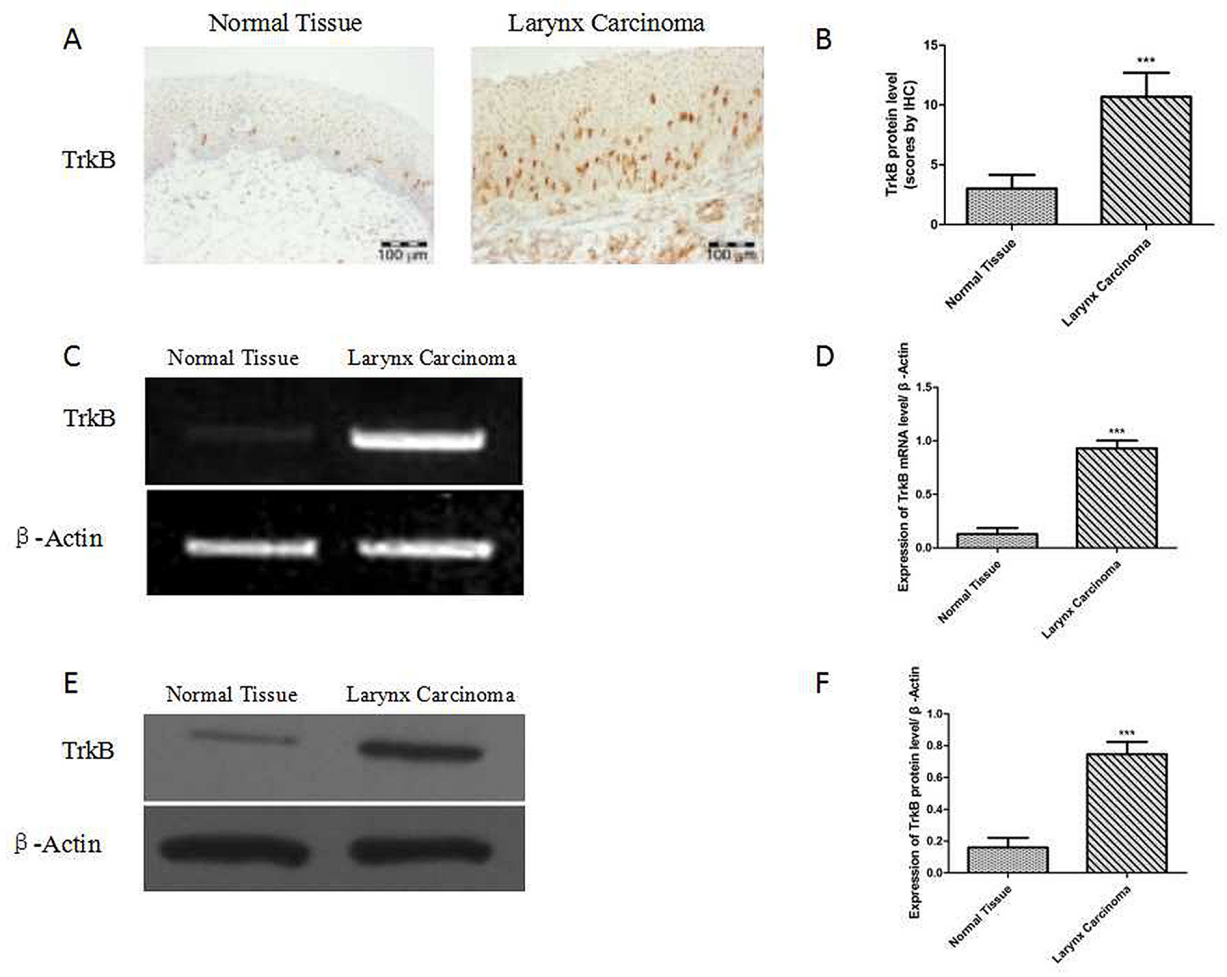
Elevated TrkB expression in laryngeal cancer tissues and laryngeal cancer cells **(A)** Immunohistochemical staining for TrkB in laryngeal cancer specimens. **(B)** Analysis of TrkB immunohistochemistry. Error bars represent standard deviations. ^***^ *P*<0.001 Vs Normal group. **(C)** and **(D)** RT-PCR analysis of expression of TrkB in laryngeal cancer specimens. **(E)** and **(F)** Western blotting analysis of TrkB expression. Error bars represent standard deviations. ^***^ *P*<0.001 Vs Normal group.

69 samples of laryngeal cancer and 32 samples of tumor-adjacent normal laryngeal tissues were used to detect the expression level of TrkB by immunohistochemistry. It showed that the expression of TrkB was significantly increased in laryngeal cancer than that in tumor-adjacent normal tissue (Figures 1A and B). The RT-PCR and western blotting analysis also exhibited that the expression of TrkB in laryngeal cancer specimens was higher than that of normal tissue (Figure 1C-1F).

### TrkB plays a functional role in laryngeal cancer cell migration

**Figure 2:**
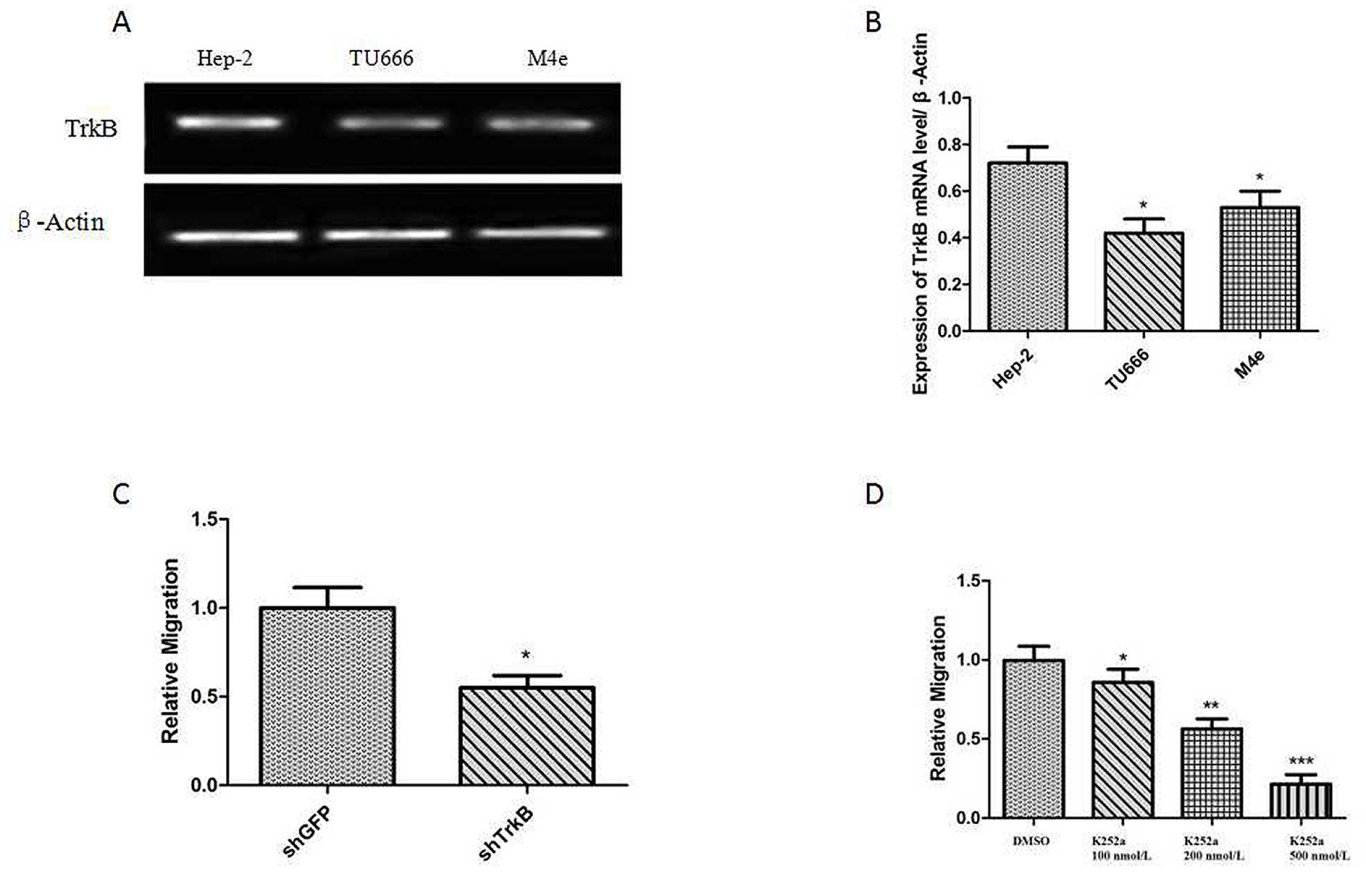
TrkB is required for laryngeal cancer cells migration **(A)** RT-PCR analysis of expression of TrkB in Hep-2 cells, TU686 cells and M4e cells. **(B)** Analysis of TrkB expression. Error bars represent standard deviations. ^*^ *P*<0.05Vs Hep-2 cells. **(C)** Transwell assays showing migration of the indicated cell lines expressing shTrkB toward conditioned media, ^***^ *P*<0.001 Vs shGFP group. **(D)** Transwell assays showing migration of the indicated cell lines treated with K252a, Error bars represent standard deviations. ^***^ *P*<0.001, ^**^ *P*<0.01, ^*^ *P*<0.05 Vs shGFP group.

Firstly, the western blotting analysis showed that the expression level of TrkB in Hep-2 was higher than that in other laryngeal tumor cell lines, including TU686 cells and M4e cells. Thus, Hep-2 cell line was used in the following studies. Secondly, Transwell assays showed that the down-regulation of TrkB expression could inhibit the migration of Hep-2 cells (Figure 2C). Subsequently, Hep-2 cells were treated with different dose of TrkB inhibitor (K252a) and the results suggested that the migration of Hep-2 cells was inhibited by K252a with a dose-dependent manner (Figure 2D) and the most significant inhibition of migration was observed at the dose of 500 nmol/L K252a. All of these results suggested that TrkB played a functional role in the migration of laryngeal cancer cell.

### TrkB regulates PI3K/AKT pathway activity via c-Src

**Figure 3:**
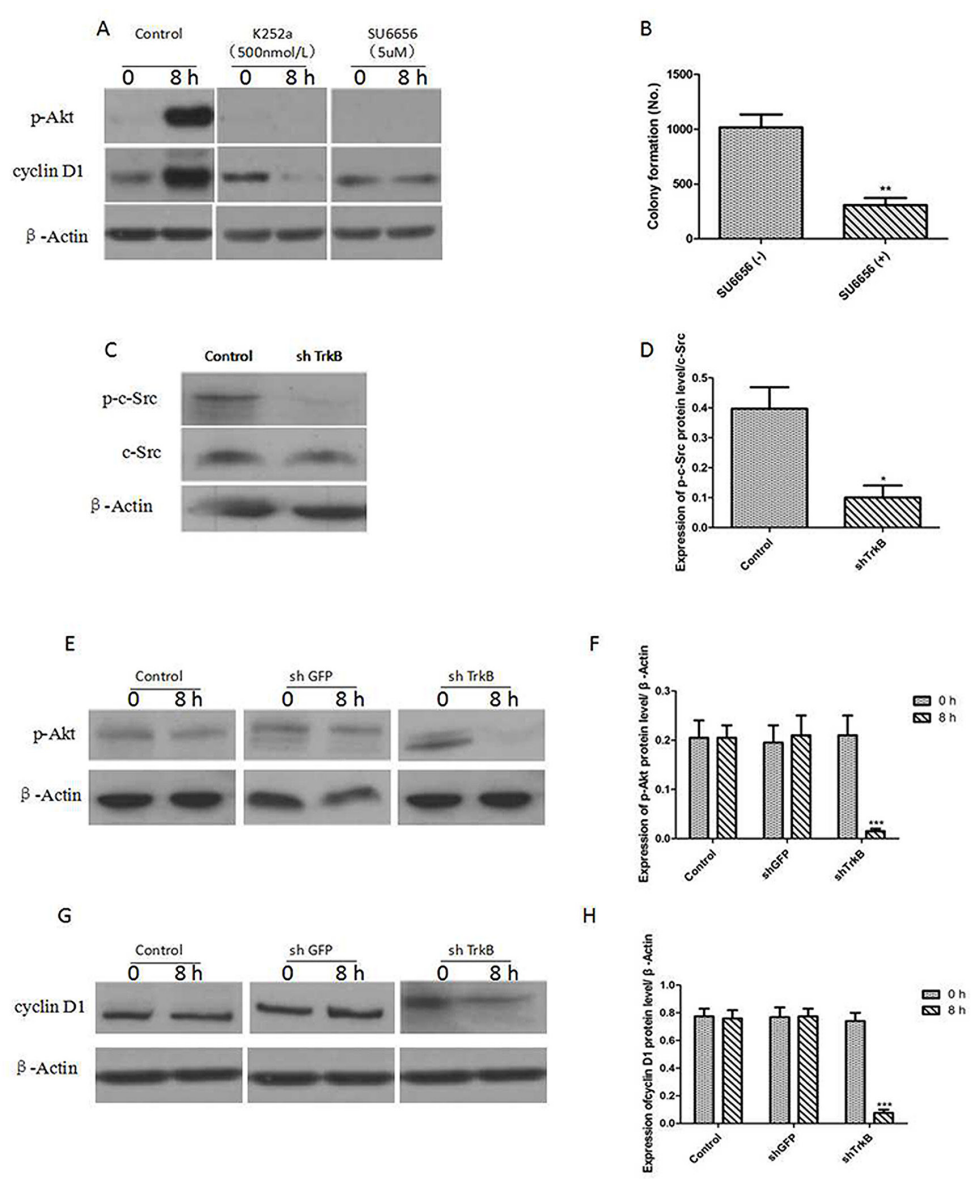
TrkB induces activation of the PI3K/AKT pathway via c-Src activation **(A)** Western blot analysis of expression of phospho-AKT and cyclin D1 in Hep-2 cells treated with 500 nmol/L K252a or 5 μM SU6656. **(B)** Colony formation assay of Hep-2 cells treated with 5 μM SU6656, Error bars represent standard deviations. ^**^ *P*<0.01Vs SU6656(-) group. **(C)** Western blot analysis of expression of p-c-Src and c-Src in Hep-2 control-shRNA or TrkB-shRNA cells. **(D)** Analysis of p-c-Src expression. Error bars represent standard deviations. ^*^ *P*<0.05 Vs control group. **(E)** Western blot analysis of expression of p-AKT in Hep-2 control-shRNA or TrkB-shRNA cells. **(F)** Analysis of p-AKT expression. Error bars represent standard deviations. ^***^ *P*<0.001 Vs shTrkB 0h group. **(G)** Western blot analysis of expression of cyclin D1 in Hep-2 control-shRNA or TrkB-shRNA cells. **(H)** Analysis of cyclin D1 expression. Error bars represent standard deviations. ^***^ *P*<0.001 Vs shTrkB 0h group.

Figure 3A showed that the expression of phosphorylated AKT (p-AKT) and cyclin D1 were significantly increased by overexpression of TrkB and pre-treatment with K252a (500 nmol/L), an inhibitor of TrkB tyrosine kinases, and SU6656 (5 μM), an inhibitor of c-Src tyrosine kinases, markedly reduced the expression of p-AKT and cyclin D1. Figure 3B showed that SU6656 (5 μM) could significantly suppressed the colony formation of Hep-2 cells. We next examined the expression of c-Src in Hep-2 control-shRNA or TrkB-shRNA cells. c-Src phosphorylation (p-c-Src) levels were significantly decreased in Hep-2 TrkB-shRNA cells, suggesting that TrkB activated c-Src in these cells (Figure 3C and 3D). We further found that AKT and cyclin D1 expression were markedly reduced in Hep-2 TrkB-shRNA cells relative to control-shRNA cells (Figure 3E-3H), indicating that TrkB could regulate the activation of AKT.

### TrkB regulates metastasis cascade through the inhibition Runx3 and Keap1

**Figure 4:**
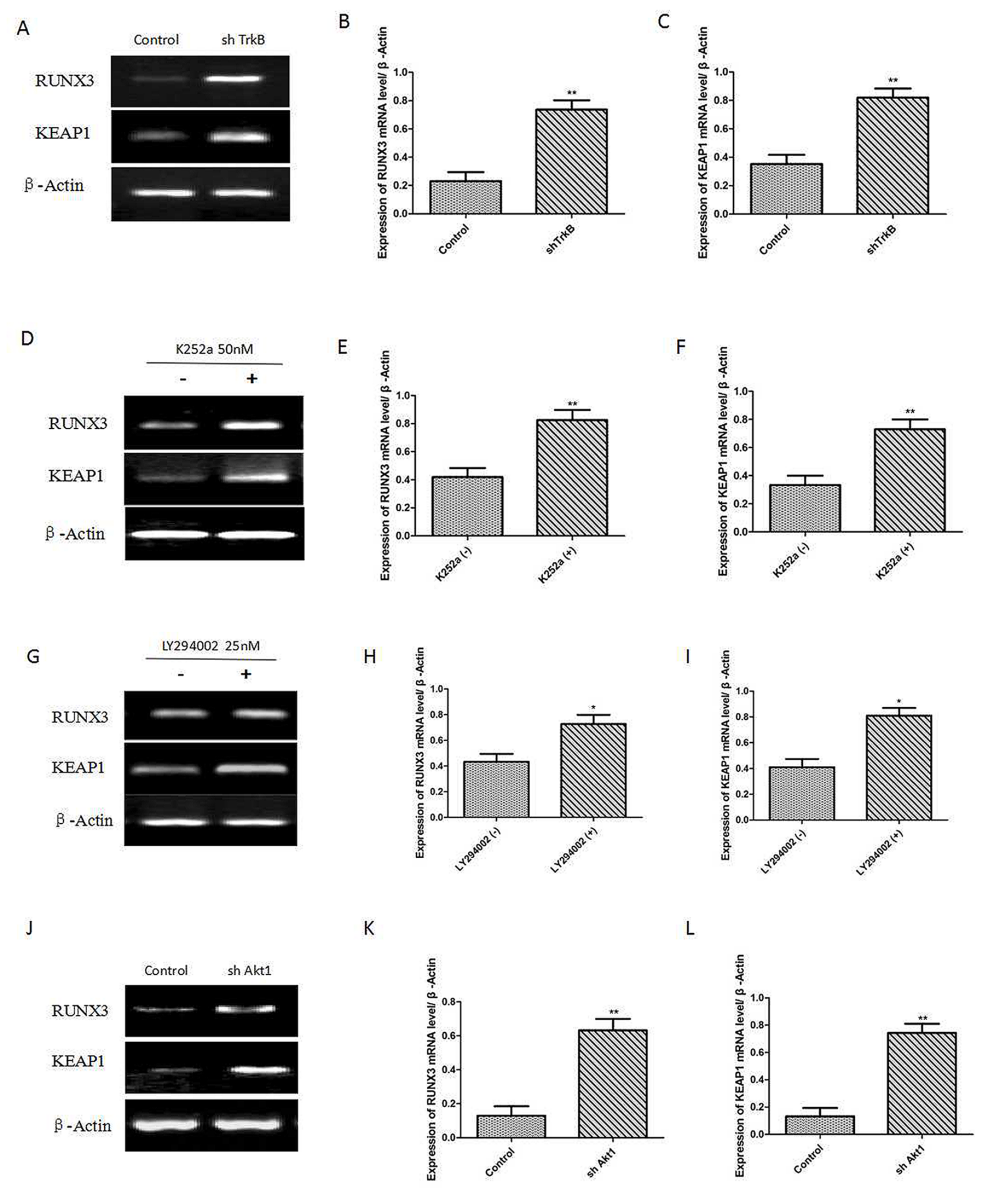
TrkB represses RUNX3 and KEAP1 expression through AKT **(A)** RTPCR analysis of Keap1 and Runx3 expression in Hep-2 control-shRNA or TrkB-shRNA cells. **(B)** and **(C)** Analysis of Keap1 or Runx3 mRNA expression. Error bars represent standard deviations. ^**^ *P*<0.01 Vs control group. **(D)** RT-PCR analysis of Keap1 and Runx3 expression in Hep-2 with or without K252a (500 nmol/L) treatment. **(E)** and **(F)** Analysis of Keap1 or Runx3 mRNA expression. Error bars represent standard deviations. ^**^ *P*<0.01 Vs K252a (-) group. **(G)** RT-PCR analysis of Keap1 and Runx3 expression in Hep-2 with or without LY294002 (PI3K inhibitor) treatment. **(H)** and **(I)** Analysis of Keap1 or Runx3 mRNA expression. Error bars represent standard deviations. ^**^ *P*<0.01 Vs LY294002 (-) group. **(J)** RT-PCR analysis of Keap1 and Runx3 expression in Hep-2 with or without shAkt1 treatment. **(K)** and **(L)** Analysis of Keap1 or Runx3 mRNA expression. Error bars represent standard deviations. ^**^ *P*<0.01 Vs control group.

We have demonstrated that c-Src activation by TrkB induces activation of the PI3K/AKT and cyclin D1 expression. To investigate the relationship between Runx3 or Keap1 and TrkB-induced tumorigenesis, we first examined whether the expression of TrkB influences Runx3 and Keap1 expression. We found that Runx3 and Keap1 mRNA expression were significantly increased in Hep-2 TrkB-sh RNA cells (Figure 4A-4C). We also found that pretreatment with K252a markedly increased Runx3 and Keap1 mRNA expression (Figure 4D-4F). We further examined whether TrkB regulates AKT activation led to reduction in Runx3 and Keap1 expression, pretreatment with the inhibition of AKT activation by LY294002 in Hep-2 cells significantly increased Runx3 and Keap1 mRNA expression (Figure 4G-4I). We further inhibited AKT activation to elucidate whether the inhibition of Runx3 and Keap1 mRNA expression in Hep-2 cells was due to transcriptional repression by AKT. Really, expression of Runx3 and Keap1 was markedly increased in Hep-2 AKT1-shRNA cells than control-shRNA cells (Figure 4J-4L).

### TrkB induces EMT through activation of the PI3K/AKT pathway

**Figure 5:**
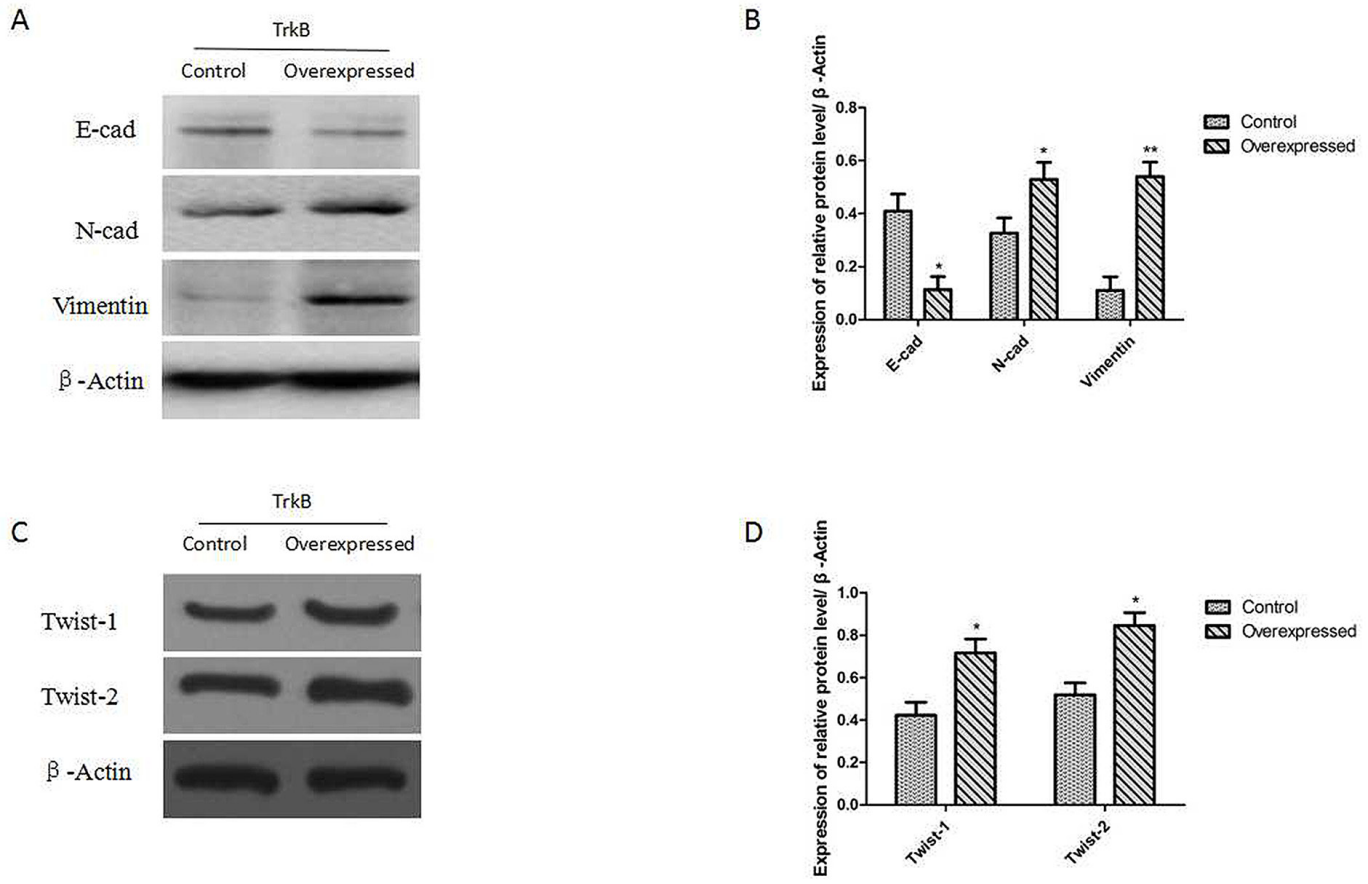
TrkB induces EMT via up-regulation of Twist-1 and Twist-2 expression **(A)** Western blot analysis of the expression of E-cadherin, N-cadherin, and Vimentin proteins in Hep-2-TrkB cells. **(B)** Analysis of E-cadherin, N-cadherin, and Vimentin expression. Error bars represent standard deviations. ^*^P<0.05, ^**^ *P*<0.01 Vs control group, respectively. **(C)** Western blot analysis of the expression of Twist-1 and Twist-2 proteins in Hep-2-TrkB cells. **(D)** Analysis of E-cadherin, N-cadherin, and Vimentin expression. Error bars represent standard deviations. ^*^P<0.05 Vs control group, respectively.

We examined whether expression of TrkB was able to promote an EMT in Hep-2 cells. The results showed that the expression of proteins E-cadherin (E-cad) was down-regulated after TrkB overexpression and the expression of proteins of N-cadherin and Vimentin were up-regulated in Hep-2-TrkB cells (Figure 5A and 5B). We further examined whether TrkB regulates the expression of Twist-1 and Twist-2. Indeed, expression of Twist-1 and Twist-2 was markedly increased in TrkB overexpressed Hep-2 cells than control cells (Figure 5C and 5D).

### TrkB markedly promotes tumor growth and metastasis *in vivo*

**Figure 6:**
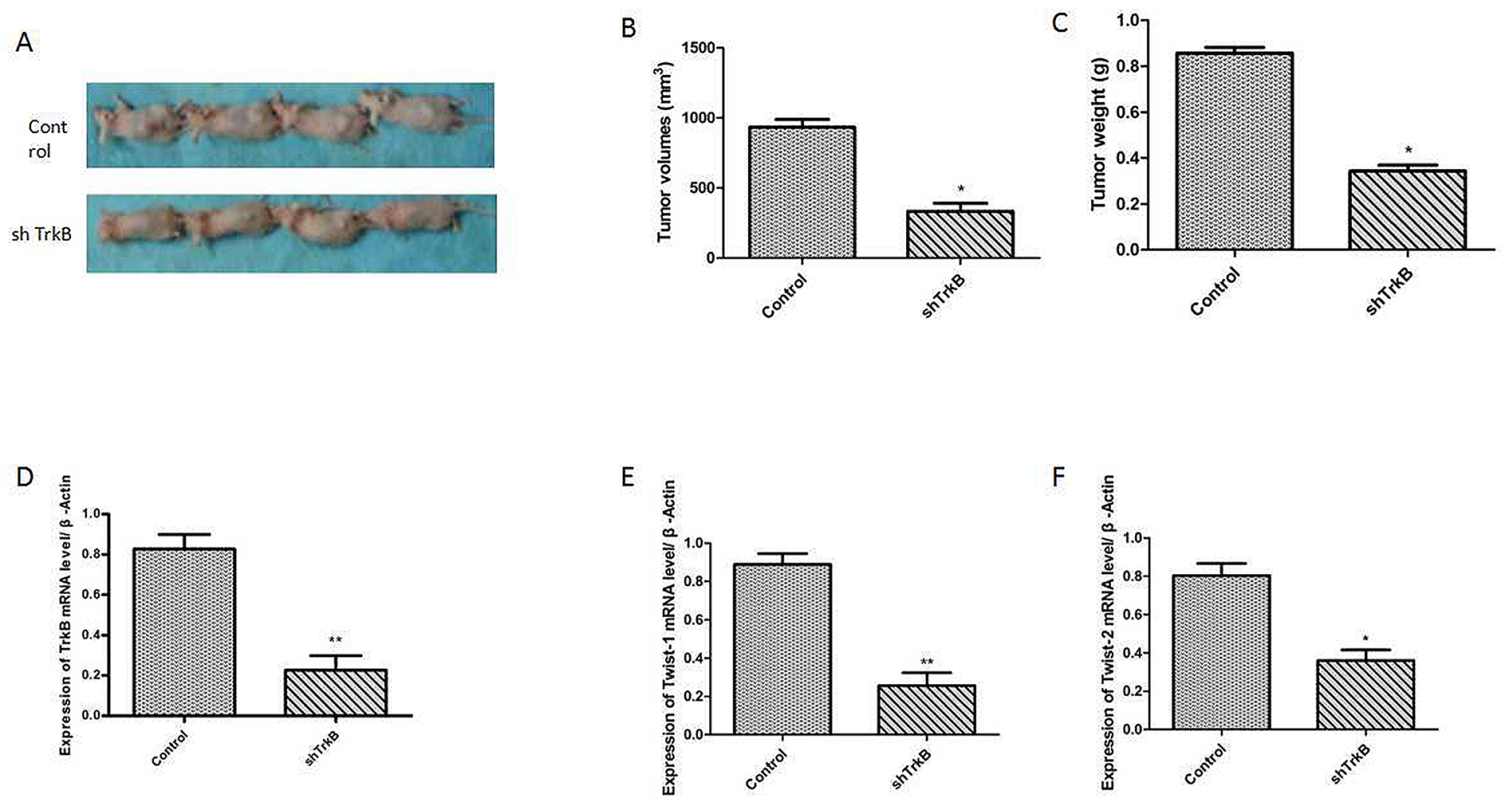
TrkB markedly pomote tumor growth and metastasis *in vivo* **(A)** Tumor formation by Hep-2 control-shRNA or Hep-2 TrkB-shRNA cells. 1.0 × 10^5^ cells were implanted in nude mice (n =10 mice/group). **(B)** Analysis of tumor volume. Error bars represent standard deviations. ^*^ *P*<0.05 Vs control group. **(C)** Analysis of tumor weight. Error bars represent standard deviations. ^*^P<0.05 Vs control group. **(D)** Analysis of TrkB mRNA expression. Error bars represent standard deviations. ^**^P<0.01 Vs control group. **(E)** Analysis of Twist-1 mRNA expression. Error bars represent standard deviations. ^**^P<0.01 Vs control group. **(F)** Analysis of Twist-2 mRNA expression. Error bars represent standard deviations. ^*^P<0.05 Vs control group.

**Figure 7:**
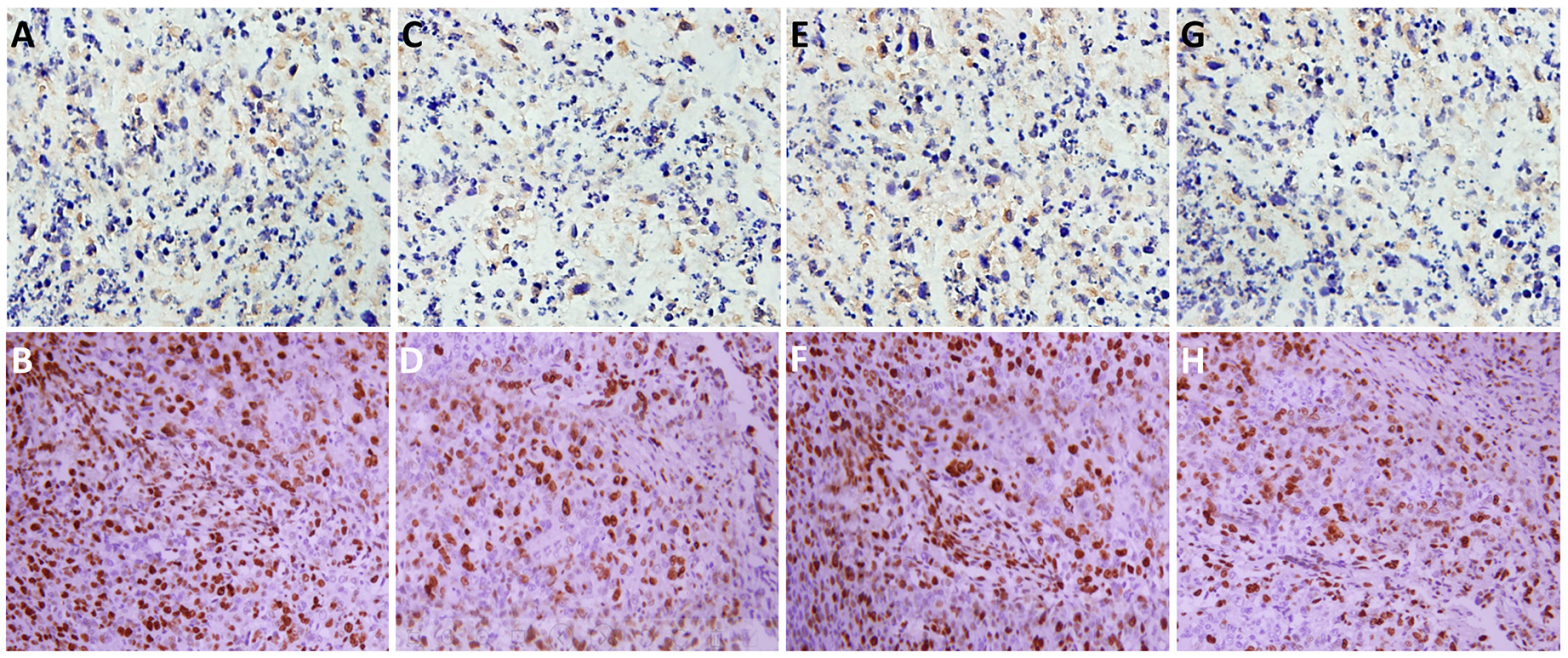
The expressions of TrkB, cyclinD1, AKT, E-cadherin in the xenograft tumor tissues **(B, D, F, H, respectively)** and normal laryngeal tissues **(A, C, E, G, respectively).**

To investigate the role of TrkB in tumor formation, Hep-2 or Hep-2-shTrkB cells were injected into of BALB/ c mice to establish tumor animal mode. After 30th days, the tumor volume and weight in Hep-2-shTrkB groups were significantly smaller than that in control group, while the inhibitory rate was markedly higher than control group (Figure 6A-6C). We next examined the expression of Twist-1 and Twist-2 in Hep-2-shTrkB group or control group, Twist-1 and Twist-2 mRNA levels were significantly decreased in Hep-2 TrkB-shRNA group (Figure 6D-6F). Figure 7 showed that the expressions of TrkB, cyclinD1, AKT, E-cadherin were also significantly enhanced in the xenograft tumor tissues comparing with those of normal laryngeal tissues.

## DISCUSSION

Laryngeal cancer is one of the most common malignant tumors of the head and neck, accounting for about 1% to 5% of the systemic tumors in our body, and the pathological type of squamous cell carcinoma, accounts for 93% to 99% of all laryngeal cancel higher incidence of laryngeal cancer is in northern China, and the age of onset is 50 to 60 years old and male with a high incidence than women [17]. In recent years, with industrial pollution increased, the incidence of laryngeal cancer has a clear upward trend. Comprehensive treatment of laryngeal cancer include surgery, application of anticancer drug therapy, radiation therapy and gene therapy [18]. With the continuous advancement of diagnosis and treatment, patients with laryngeal cancer after repair function, quality of life and 5-year survival rates are greatly improved, but local recurrence and metastasis is still the main reason for laryngeal cancer patients died within 5 years and the treatment of patients with advanced laryngeal cancer is still not satisfactory [19]. On the other hand, minimally invasive surgery also requires a correct estimate of laryngeal cancer early diagnosis and prognosis of laryngeal cancer, in order to minimize the trauma to retain the function of the patient’s throat. Exploring relevant factors related to the incidence of laryngeal cancer, investigating more effective indicators for early and accurately confirming pathological types, understanding the molecular mechanisms of tumorigenesis and development of laryngeal cancer, and determining the reasonable treatment strategies have become four important points for clinical researches.

In this study, we show that TrkB is frequently overexpressed in highly metastatic laryngeal cancer cell lines and clinical laryngeal cancer samples. Furthermore, TrkB expression was significantly up-regulated in laryngeal cancer samples in comparison with normal laryngeal tissue. Activation of the PI3K/AKT pathway through TrkB-mediated c-Src activation induced tumorigenic and metastatic potential of laryngeal cancer.

Tumor metastasis is an important reason causing tumor morbidity and mortality. Several studies have shown that TrkB play an essential role in invasion and metastasis of various tumor cells [20, 21]. In this study, we found that knockdown of TrkB could inhibit cell migration, and treatment with the Trk inhibitor K252a would reduce the p-AKT level in laryngeal cancer. Reduction of TrkB signaling similarly decreased colony formation, and tumor xenograft growth of Hep-2. It indicated that TrkB was a key regulator for laryngeal cancer cell migration.

Studies have shown that PI3K/AKT signaling pathway involved in development of human malignant tumors, and the pathway is related with tumor proliferation, apoptosis, metastasis, invasion and other cellular processes [22, 23]. PI3K/AKT signaling pathway is becoming a treatment target for many kinds of cancer, including nasopharyngeal carcinoma, pancreatic cancer, thyroid cancer, lung cancer, gastric cancer, bladder cancer and breast cancer [24, 25]. Studies have confirmed that abnormal activation of PI3K/AKT signal pathway is associated with the occurrence of laryngeal squamous carcinoma [26].

c-Src played essential roles in multiple aspects of tumor progression, including proliferation, disruption of cell/cell contacts, migration, invasiveness, resistance to apoptosis, and angiogenesis [27]. Moreover, c-Src could activate PI3K/AKT and the MEK/ERK cascades, which indicated that TrkB regulated tumor progression by activation of PI3K/AKT signaling pathway via regulation of c-Src [28]. In the present study, we found that activation of the PI3K/AKT pathway through TrkB-mediated c-Src activation induced tumorigenesis and metastasis of laryngeal cancer. Kim et al. [29] suggested that TrkB could activate AKT by directly binding to c-Src leading to PI3K/AKT pathway-mediated tumor metastasis and EMT program. TrkB was a key regulator of PI3K/AKT and JAK/STAT signal pathway-mediated EMT program, which played a key role during early steps of invasion and metastasis of epithelial malignancies, [30]. EMT involves an orchestrated series of events in which epithelial cells lose polarity, cell-cell contacts and undergo dramatic remodeling of the cytoskeleton which enables carcinoma cells to migrate through an extracellular environment and settle in distinct areas to form metastasis [31, 32]. Overexpression of TrkB in Hep-2 led to EMT-like state dependent on the Twist-1 and Twist2. Our data showed that TrkB could induce EMT via increasing the expression of EMT related transcription factors such as Twist-1 and Twist-2. Moreover, the expressions of TrkB, cyclinD1, AKT, E-cadherin were also significantly enhanced in the xenograft tumor tissues, further confirming that TrkB signaling pathway played an essential role in the tumorigenesis and metastasis of laryngeal cancer.

In conclusion, TrkB are overexpressed in laryngeal cancer, and TrkB signaling is involved in tumorigenesis and metastasis of laryngeal cancer. It indicates that TrkB pathway may be a promising target for future intervention strategies to prevent tumor metastasis, EMT program in laryngeal cancer. The future study should focus on the targeting regulation of TrkB pathway via non-coding RNA which may provide a novel therapeutic plan for laryngeal cancer.

## MATERIALS AND METHODS

### Cell lines and main reagents

Human laryngeal cancer cell lines (Hep-2, TU686 and M4e) were obtained from the Cell Bank of the Chinese Academy of Sciences (Shanghai, China) and were maintained as previously described [33–35]. Briefly, laryngeal cancer cells were cultured in a 1:1 mixture of Ham's F12 and Dulbecco's modifed Eagle's medium (DMEM) with 10% fetal bovine serum (FBS), 100 U penicillin and 100 mg/ml streptomycin. The cells were maintained at 37°C in a humidified atmosphere containing 5% CO_2_. The inhibitors of protein kinase (K252a and SU6656) and PI3K inhibitor (LY294002) was purchased from Calbiochem (San Diego, California, USA).

### Human laryngeal cancer samples

Fresh-frozen human laryngeal cancer samples and tumor-adjacent normal laryngeal tissues were obtained from the Affiliated Hospital of Southwest Medical University. The subject was approved by the institutional review board and the ethics committee of the Affiliated Hospital of Southwest Medical University (K2013166) and all patients have signed informed consents.

### Immunohistochemistry

The paraffin sections of laryngeal cancer were deparaffinized and rehydrated routinely. The sections were immersed with 3% H_2_O_2_ and 10% goat serum and they were subsequently incubated overnight with a primary antibody for TrkB (sc-8316, Santa Cruz Biotechnology, Santa Cruz, CA, USA, 1:100) at 4°C. The samples were incubated with HISTOFINE simple stain MAX-PO (R) (Nichirei, Tokyo, Japan) and finally visualized by 3,3’diaminobenzidine (DAB) with hematoxylin-counterstain.

### Plasmid tranfections

Short-hairpin RNA (shRNA) constructs targeting TrkB (shTrkB) and AKT1 (shAKT1) and pLKO were obtained from Sigma-Aldrich (St. Louis, MO, USA) and introduced into cells via retroviral infection or with lipofectamine2000 reagent according to the manufacturer’s protocol. shRNA that did not match any known human cDNA was used as a control. For TrkB overexpression, pcDNA3.1 plasmids containing the full length cDNA sequences for human TrkB was stably transfected into Hep2 cells.

### Western blotting

Western blotting was performed as previous reported [36, 37]. Briefly, Hep-2 cells were grown in the mid-log phase, washed with phosphate buffered saline and lysed for 30 min on ice. The lysis buffer with pH 6.8 contained 10% glycerol, 5% 2-mercaptoethanol, 2% sodium dodecyl sulfate (SDS), 62.5 mmol/L Tris-HCl. The SDS–PAGE analysis was performed and membranes were incubated overnight at 4°C with antibodies directed against the indicated proteins. The antibodies were obtained from the following companies: anti-TrkB, anti-β-actin, anti-p-Akt, anti-cyclin D1, anti-p-s-Src and anti-c-Src were purchased from Santa Cruz Biotechnology (Santa Cruz, CA, USA); anti-RUNX3 and anti-Keap1 were purchased from Cell Signaling Technology (Danvers, MA, USA); anti-E-cadherin, anti-N-cadherin, anti-vimentin, anti-Twist-1 and anti-Twist-2 were purchased from BD Biosciences (San Jose, CA, USA). Membranes were washed, incubated with the appropriate secondary antibodies and exposed with the ECL chemiluminescent substrate kit (Pierce, Rockford, IL, USA). Images were analyzed with ImagePro (Media Cybernetics, Bethesda, MD, USA) and densitometry data were analyzed by using either conventional Student’s t-test. Results are reported as mean ± SD. A P-value <0.05 was considered significant and all were two-tailed.

## RT-PCR

RT-PCR was performed as previous reported [38]. Briefly, the extraction of total RNA was conducted by using the High Pure Isolation Kit (Roche Diagnostics Gmbh, Mannheim, Germany). The quality and quantity of extracted RNA was analyzed by using Nanodrop (ND-1000) spectrophotometer (NanoDrop Technologies, Inc., Wilmington, DE) at the absorbance ratio of 260 and 280 nm. The reverse transcription reaction was conducted with the Quantitect Reverse Transcription Kit (Qiagen, Hilden, Germany) according to the manufacturer’s protocol. PCR were performed with SYBR Premix EX Taq II (Takara Bio, Shiga, Japan) on a DNA Engine Opticon 2 system (MJ research, Waltham, MA, USA). The amount of each target gene was normalized to the level of β-actin. The primers used were as follows:

TrkB: forward: 5-GGAAAAGCAAAAACCCTGTCTAGA-3;
reverse: 5-TGTAGCATCACTTCCTGCCATT-3
β-actin: forward: 5-TTGCCCCGAGGCTCTCTT-3;
reverse: 5-AGTTGAAGGTGGTCTCGTGGAT-3.

After a reverse transcription reactions at 55°C for 30 minutes, there was an initial 10-minute denaturation at 95°C followed by the 40 cycles run consists of a 15-second denaturation step at 94°C and an annealing step at 60°C for 30 seconds, and extension step at 68°C for 1 minute. The density of bands was determined by using Un-Scan-It gel Software (Silk Scientific Corp., Orem, UT) and statistical analysis was performed by utilizing either conventional Student’s t test. The PCR results were averaged with three independent experiments, which were used in the statistical analysis.

### *In vivo* xenograft tumor model

Five-week-old male athymic nude mice (BALB/c nu/nu) were purchased from Chengdu Dashuo Biological Technology Co. Ltd and acclimated for two weeks. All animal procedures were approved by the Animal Care and Use Committee at animal experimental center of Southwest Medical University. The animal experiment was also approved by the institutional review board and the ethics committee of the Affiliated Hospital of Southwest Medical University (K2013167), Hep-2 cells transfected with TrkB-targeting shRNA or non-targeting control shRNA were subcutaneously implanted into flank regions (1×10^5^ cells in PBS per mouse) of the nude mice (n=10 in each). Immunohistochemistry of TrkB, cyclinD1, AKT, E-cadherin were also performed in the xenograft tumor tissue. The primary antibodies for TrkB (sc-8316, 1:100), cyclinD1 (sc-4074 WB, 1:100), AKT (sc-56878, 1:100), E-cadherin (sc-52327, 1:100) were purchased from Santa Cruz Biotechnology (Santa Cruz, CA, USA). The procedures were similar with the part of immunohistochemistry. Briefly, the paraffin sections of xenograft laryngeal cancer were deparaffinized, rehydrated and immersed with 3% H_2_O_2_ and 10% goat serum. Subsequently, the sections were incubated overnight with primary antibodies, followed by incubation with HISTOFINE simple stain MAX-PO (R) (Nichirei, Tokyo, Japan) and visualization by DAB with hematoxylin-counterstain.

### Statistical analysis

All data were analyzed by means of SPSS 18.0 (IBM Software, Armonk, NY, USA) and were shown as the mean ± standard deviation (SD). Student’s t-test was used for the comparison of mean values between two groups. A value of P< 0.05 was considered significant.

## Abbreviations

AKT: serine/threonine-protein kinases
BDNF: brain-derived neurotrophic factor
DAB: 3,3’diaminobenzidine
DMEM: Dulbecco's modifed Eagle's medium
ERK: extracellular signal-regulated kinase
EMT: epithelial-to-mesenchymal transition
FBS: fetal bovine serum
Keap1: Kelch like ECH associated protein1
MEK: mitogen-activated protein signal-regulated kinase
NT4: neurotrophin 4
PI3K: phosphoinositide 3-kinase
p-AKT: phosphorylated AKT
RT-PCR: reverse transcription polymerase chain reaction
Runx3: human runt-related transcription factor 3
Src: steroid receptor coactivator
ShRNA: short-hairpin RNA
SDS: sodium dodecyl sulfate
TrkB: tropomyosin-related kinase B receptor
